# Metabolic Adaptation and Protein Complexes in Prokaryotes 

**DOI:** 10.3390/metabo2040940

**Published:** 2012-11-16

**Authors:** Beate Krüger, Chunguang Liang, Florian Prell, Astrid Fieselmann, Andres Moya, Stefan Schuster, Uwe Völker, Thomas Dandekar

**Affiliations:** 1 Department of Bioinformatics, Biocenter, Am Hubland, University of Würzburg, 97074 Würzburg, Germany; Email: beate.krueger@biozentrum.uni-wuerzburg.de (B.K.), liang@biozentrum.uni-wuerzburg.de (C.L.); Florian.Prell@stud-mail.uni-wuerzburg.de (F.P.); astrid.fieselmann@uni-wuerzburg.de (A.F.); 2 Unidad Mixta de Investigación en Genómica y Salud CSISP-UVEG, University of València José Beltrán 2, 46980 Paterna, Valencia, Spain; Email: andres.moya@uv.es (A.M.); 3 Cavanilles Institut on Biodiversity and Evolutionary Biology, University of València José Beltrán 2, 46980 Paterna, Valencia, Spain; 4 Department of Bioinformatics, Friedrich-Schiller-University Jena, Ernst-Abbe-Platz 2, 07743 Jena, Germany; Email: stefan.schu@uni-jena.de (S.S.); 5 Department of Functional Genomics, Interfaculty Institute for Genetics and Functional Genomics, Ernst-Moritz-Arndt-University Greifswald, Friedrich-Ludwig-Jahn-Straße 15a, 17487, Greifswald, Germany; Email: voelker@uni-greifswald.de (U.V.); 6 European Molecular Biology Laboratory, Meyerhofstr. 1, 69012 Heidelberg, Germany

**Keywords:** metabolites, protein complexes, prokaryotes, crowding, channeling, *S. aureus*, *E. coli*

## Abstract

Protein complexes are classified and have been charted in several large-scale screening studies in prokaryotes. These complexes are organized in a factory-like fashion to optimize protein production and metabolism. Central components are conserved between different prokaryotes; major complexes involve carbohydrate, amino acid, fatty acid and nucleotide metabolism. Metabolic adaptation changes protein complexes according to environmental conditions. Protein modification depends on specific modifying enzymes. Proteins such as trigger enzymes display condition-dependent adaptation to different functions by participating in several complexes. Several bacterial pathogens adapt rapidly to intracellular survival with concomitant changes in protein complexes in central metabolism and optimize utilization of their favorite available nutrient source. Regulation optimizes protein costs. Master regulators lead to up- and downregulation in specific subnetworks and all involved complexes. Long protein half-life and low level expression detaches protein levels from gene expression levels. However, under optimal growth conditions, metabolite fluxes through central carbohydrate pathways correlate well with gene expression. In a system-wide view, major metabolic changes lead to rapid adaptation of complexes and feedback or feedforward regulation. Finally, prokaryotic enzyme complexes are involved in crowding and substrate channeling. This depends on detailed structural interactions and is verified for specific effects by experiments and simulations.

## 1. Introduction

In cells, proteins often occur together with other proteins in protein complexes. The proteins in these complexes often interact to fulfill their function. In this review, the aim is to explore how complexes interact from the aspect of systems biology, how they adapt to changes in the environment and how this is connected to metabolism and its regulation, including crowding and channeling effects. 

In general, for a comprehensive view on protein complexes, a large amount of data, integrated models and comparative biology on various species is required [1,2,3,4,5,6,7]. This is now possible as, for the first time, there is sufficient data for a comprehensive systems-level view on how metabolic adaptation is accomplished [1]. In this review, we illustrate how protein complexes help to establish order and improve adaptation in the prokaryotic cell, particularly in regards to metabolism.

Large-scale data are critical for a comprehensive view on protein complexes and metabolic adaptation, hence we first provide a view on different large-scale screening studies on protein complexes in prokaryotes [3,4,5,6,7]. Each of these studies brought up new complexes and there are certainly more surprises in stock. These studies classified protein complexes and are also a useful pointer to detailed catalogs of prokaryotic proteins, interactions and protein complexes and connections to metabolism. An interesting insight are super-complexes connecting complexes. They organize the cell in a factory-like fashion to optimize protein production and metabolism [4]. Here, central components are conserved between different prokaryotes [8].

We then look at the connections between metabolic adaptation and protein complexes. Proteins such as trigger enzymes display in a condition-dependent fashion two (or more) different functions by participating in several complexes [9]. Intracellular pathogens utilize different metabolites in their respective niche with rapid adaptation of metabolism and involved protein complexes [1]. Prokaryotic regulatory strategies ensure optimal mRNA and protein half-life as well as optimal growth under different environmental and niche conditions [10].

A system-wide, global view on prokaryotic protein complexes shows rapid adaptation supported by system shifts promoted by protein switches (e.g., central transcription regulators) and feedforward and feedback loops [7]. Though some examples may be charted, it is clear that we need more studies and more data to really understand system-wide regulation of prokaryotic protein complexes.

Protein complexes are also very effective on a molecular structure level in providing a molecular framework in the prokaryotic cell, e.g., for metabolism [2]. As crowding and substrate channeling have been studied for a long time, we also provide recent data from experiments and simulations for these effects mediated by protein complexes.

Rapid growth and swift adaptation are hallmarks of the prokaryotic lifestyle; protein complexes help to organize metabolism and adaptation to efficiently achieve both goals [6].

## 2. Results and Discussion

### 2.1. Protein Complexes in Central Metabolism

**Figure 1:**
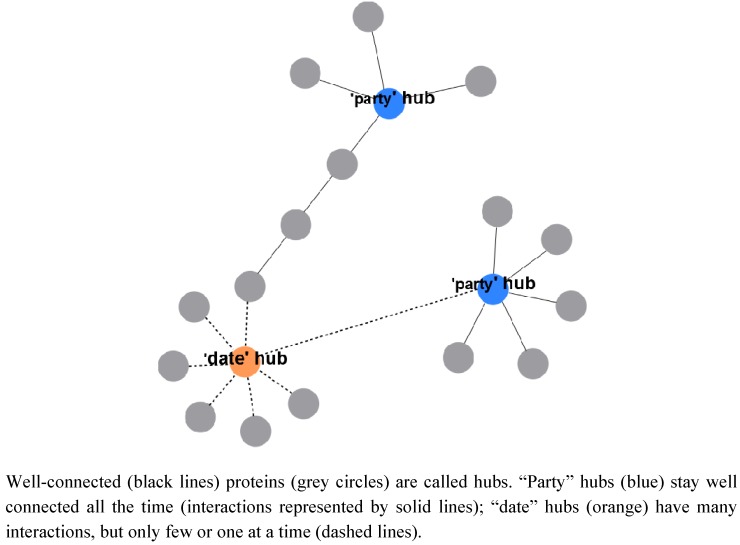
Proteins and complexes.

To understand how protein complexes are organized and how metabolism is connected to these, one has to look first at suitable large-scale studies on protein complexes to have sufficient data. In general, proteins which are well connected in protein complexes are called “hubs” and one can distinguish between permanently well-connected “party” hubs and proteins that have many connections, but specific interactions are reserved for small time slots, known as “date” hubs (Figure 1, [11]). Furthermore, for all protein complexes, there are central core complexes and accessory components outside. The accessory components may either be adaptor proteins for interactions, occurring as shared components in various complexes, or are only accessory components for one specific complex [10].

Structural considerations for the network topology include consideration of network centrality. Thus, general metabolic pathways are connected by short pathways and currency metabolites are well buffered to achieve optimal balancing of the network. Whether such networks are truly “small world”—like [12] or not [13] is still a matter of debate. Small world-like behavior often reflects agglomeration, and evolutionary forces drive such processes (e.g., pathway duplication, pathway recruitment *etc.*, [14]), thereby enhancing exactly this growth type, including metabolic enzyme complexes. There is also the concept of a large central component with several smaller bystander networks and a comparatively high number of singletons [15]. This is again a typical finding from interactomics [16], but partly reflects true effects of evolutionary forces at work. However, partly also natural limitations of knowledge (most data instances can be connected, so we get a large central component, and similar reasoning for other subnetworks) becomes a problem. 

Selection optimizes metabolic networks in bacteria further. For instance, metabolic pathways in bacteria are organized to be optimally switched by central transcription factors and, in this respect, there is certainly a selection for optimal control. Controllability in different types of networks is currently a hot topic of research [17]. 

Regarding large-scale studies on prokaryotic complexes, focusing on one of the smallest bacteria known and profiting from its compact genome, Kühner *et al.* [4] used tandem affinity purification-mass spectrometry (TAP-MS) on the small Gram-positive bacterium *M. pneumoniae*. This revealed 62 homomultimeric and 116 heteromultimeric soluble protein complexes which are partly conserved across bacteria, including many novel ones. Interestingly, a third of the complexes are involved in higher levels of proteome organization. These larger multi-protein complex entities link successive steps in biological processes like a conveyor belt involving shared multifunctional components. This interesting finding of a factory-like arrangement of bacterial protein complexes churning out a maximum of proteins and processed metabolites was supported by structural analysis on 484 proteins (single particle electron microscopy, cellular electron tomograms and bioinformatical models). Thus, Kühner *et al.* [4] show details of the factory and interlinked protein complexes, including detailed structure prediction. Regarding time-dependent nuclear complexes, they found multiple regulators and regulatory interactions per prokaryotic gene, such as new noncoding transcripts. For instance, there are 89 of them in antisense configuration to known genes in *M. pneumoniae* [3]. With similar techniques, Butland *et al.* [18] analyzed *E. coli* complexes using affinity tagged proteins of 1,000 open reading frames (nearly a quarter of the genome). 648 were homogeneously purified and analyzed by mass spectrometry. The direct experimental approach revealed new interactions, as well as interactions predicted previously based on bioinformatic approaches from genome sequence or genetic data. Furthermore, looking in detail at both data sets ([3,18]) shows that many important interactions are conserved in both bacteria.

The question of conservation of prokaryotic protein complexes and their interactions was also analyzed by Parrish *et al.* [8] in the food-borne pathogen *Campylobacter jejuni* (NCTC11168). Yeast two-hybrid screens identified 11,687 interactions with 80% of all bioinformatically predicted proteins participating. Furthermore, this study places a large number of poorly characterized proteins into networks with hints about their functions. Interestingly, a number of their subnetworks are not only conserved compared to *E. coli*, but also to *S. cerevisiae*. Furthermore, biochemical pathways can be mapped on protein interaction networks. This has been shown in this study for the chemotaxis pathway *of C. jejuni*. As an application aspect, a large subnetwork of putative essential genes suggests new antimicrobial drug targets for *C. jejuni* and related organisms. In summary, this landmark study [8] nearly doubled the binary interactions described for prokaryotes at that time, and showed that many of the identified complexes are conserved in their central components between various prokaryotic organisms. 

**Figure 2:**
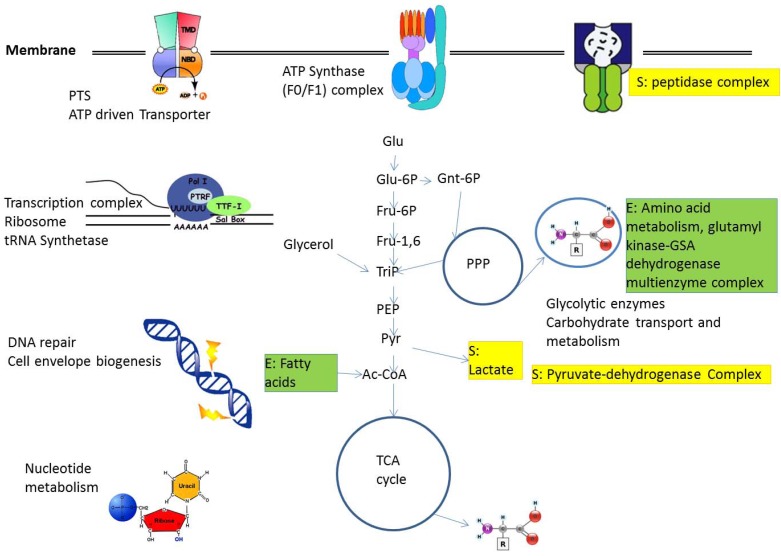
Protein complexes and their connection with metabolism. A number of central complexes are shown, giving the situation in *E. coli*, as well as implied and connected central metabolic pathways. In *S. aureus*, details of several complexes differ (*E. coli*-specific complexes not present in *S. aureus* are labeled with **E** (in green), *S. aureus*-specific complexes not present in *E. coli* are labeled with **S** in yellow, see text). For details see text. Abbreviations: Ac-Coa, acetyl-CoA; Fru-1,6, fructose-1,6-bisphosphate, Fru-6P, fructose 6-phosphate; Glu, glucose; Glu-6P, glucose 6-phosphate; Gnt-6P, 6-phospho gluconate; PEP, phosphoenolpyruvate; PPP, pentose phosphate pathway; PTS, phosphotransferase system; Pyr, pyruvate; TCA, tricarboxylic acid; TriP, triose phosphate.

Figure 2 shows a number of complexes available from these studies and found either in *E. coli* or *Staphylococcus aureus* or both as well as their connection to metabolism.

The annotation and validation of all the implied prokaryotic interaction data and protein complexes is nontrivial. One important way to achieve this is to make them accessible by a Wiki, for instance, the “SubtiWiki” or the “WikiPathways”. These Wikis provide a knowledgebase for the Gram-positive model bacterium *Bacillus subtilis* [19], or pathways in general [20]. The SubtiWiki includes the companion databases SubtiPathways [21] and SubtInteract with graphical presentations of metabolism, its regulation, as well as protein–protein interactions and complexes, and is highly recommended as an exemplary resource to study systems biology of protein complexes in bacteria.

Moreover, protein modifications have to be charted. They influence protein complex formation, and removing or adding a protein modification allows corresponding protein complexes to change with time. An important mechanism is phosphorylation. Interacting proteins may specifically bind to these protein modifications or only bind if these modifications are absent. Thus, Van Noort *et al.* [22] compared this mechanism with other posttranscriptional regulatory mechanisms in *M. pneumoniae*. This organism is particularly suited for such studies because it encodes only two protein kinases and one protein phosphatase. This fact allows an elegant identification of the protein-specific effects on the phosphorylation network using specific deletion mutants. Van Noort and co-workers also considered changes in protein abundance and lysine acetylation [22]. Introduction of the mutations did not alter the transcriptional response, but deletion of the two putative N-acetyltransferases affected protein phosphorylation, which demonstrates the cross-talk between the two posttranslational modification systems. Phosphoproteome studies were also reported for *B. subtilis*. Jers *et al.* [23] identified nine previously unknown tyrosine-phosphorylated proteins in *B. subtilis*, and the majority of these were at least *in vitro* PtkA substrates.

### 2.2. Metabolic Adaptation and Protein Complexes

For growth, intracellular model pathogens rely on different metabolic resources and exploit suitable protein complexes for their utilization and thus regulate metabolism accordingly. Therefore, comparing the wild-type *S. aureus* strain 8325 and the isogenic deletion mutants (either lacking the eukaryotic-like protein serine/threonine kinase PknB or the phosphatase Stp, [24]) remarkable differences were found. Those differences were in nucleotide metabolism and cell wall precursor metabolites, such as peptidoglycan and cell wall teichoic acid biosynthesis in *S. aureus*. This phosphatase and the kinase are also antagonistic players in central metabolism, affecting enolase, triose phosphate isomerase, fructose biphosphate aldolase, pyruvate dehydrogenase, phosphate acetyl transferase, and others.

Similarly*, S. aureus* pathogenicity potential depends on the iron status of the host [25]. Combining difference in-gel electrophoresis and mass spectrometry with multivariate statistical analyses, Friedman *et al.* [25] revealed clusters of cellular proteins responding to distinct iron-exposure conditions (iron chelation, hemin treatment), as well as genetic changes (∆*fur*). 120 proteins representing several coordinated biochemical pathways and regulons were affected by changes in iron-exposure status, for instance the heme-regulated transport system (*hrtAB*), a novel transport system. During iron starvation, pH decreased and acidic end-products accumulated so that iron was released from the host iron-carrier protein transferrin. 

Complexes may thus rapidly assemble and disassemble according to the metabolic situation. To achieve this efficiently, “moonlighting” enzymes have a hidden second function only apparent in the “moonlight”, *i.e.*, an alternative metabolic condition revealing its nonstandard function. Aconitase is a good example; with sufficient iron content, its iron-sulfur cluster is present and the enzyme catalyzes isomerization of citrate to isocitrate. However, under low iron, a hidden second activity is apparent: without an iron-sulfur cluster the enzyme binds iron-responsive elements in RNA to block translation. Such enzymes are thus found in two different complexes (e.g., metabolic complex or RNA-binding complex) and change their life from metabolism to control of gene expression in response to the availability of their substrates (“trigger enzymes”; [9]). Other enzymes have acquired a DNA-binding domain. They act as direct transcription repressors by binding DNA in the absence of substrate. Furthermore, sugar permeases of the phosphotransferase system control transcription activity by phosphorylating regulators in the absence of a specific substrate [26]. Finally, regulatory enzymes may control transcription factors by inhibitory protein–protein interactions. Duplication and subsequent functional specialization, a general motor of enzyme evolution, is also a major evolutionary pattern found here.

#### 2.2.1. Metabolic Adaptation in Intracellular Model Pathogens

In *Lysteria monocytogenes* the transcriptional regulator PrfA controls levels of pathogenicity factors and influences protein complexes and metabolic pathways, but also allows adaptation to the nutrient-poor, low-glucose environment of the cytoplasm of the host [27]. The metabolism of host and pathogen is intertwined and *L. monocytogenes* is well adapted to this nutrient-poor environment, not disturbing the balance of the host too much. Overexpressed PrfA strongly influences the synthesis of some amino acids, such as branched amino acids (Val, Ile and Leu). Degradation of glucose occurs *via* the pentose phosphate pathway. The citrate cycle is incomplete (lack of 2-oxoglutarate dehydrogenase). Oxaloacetate is formed by carboxylation of pyruvate. Furthermore, growth of *L. monocytogenes* on brain–heart infusion medium resulted in a substantial upregulation of all genes and protein complexes involved in facilitated glycerol uptake (*glpF*) and catabolism of glycerol, such as glycerol kinase (*glpK*), glycerol-3-phosphate (glycerol-3P)-dehydrogenase (*glpD*), and dihydroxyacetone kinase subunit K (*dhaK*). In contrast, genes encoding the permeases of the PEP-dependent phosphotransferase system (PTS) are not upregulated. In fact, there is downregulation of glycolysis genes and upregulation of gluconeogenesis. Glycerol may be a major carbon source for carbon metabolism in intracellular bacteria. Glucose-6P may serve as an additional carbon source whereas glucose is probably not a major substrate for intracellular *Listeria*. Important for intracellular survival and virulence is the ATP-dependent pyruvate carboxylase (PycA). Furthermore, only some amino acids are synthesized *de novo* (Ala > Asp > Glu > Ser > Thr > Val > Gly) [28]. Cofactors such as riboflavin, thiamine, biotin and lipoate are directly imported from the host cell.

For comparison, in *Shigella flexneri*, glucose uptake is downregulated and glycerol utilized in cytosolic growth within macrophages. Gluconeogenesis (*fbp* and *pps*) is upregulated. Under these conditions, *Shigellae* synthesize aromatic amino acids, GMP and thymidine, and the corresponding enzyme complexes.

In contrast, pathogenic *E. coli* strains (EIEC) utilize glucose for survival inside the host cell [1]. However, similar to the *Shigaellae*, EIEC are also more anabolically active in their intracytoplasmatic lifestyle than *Listeria*, as EIEC synthesize their own amino acids. 

Intracellular *Salmonella enterica* subsp. *enterica* serovar Typhimurium use glucose, glucose-6P and gluconate (glycolysis and Entner-Doudoroff pathway are upregulated, TCA is downregulated). In the *Salmonella* containing vacuole, glucose is preferred over glucose-6P as carbon substrate. In systemically infected mice, bacterial growth depends on a complete TCA cycle [29] and the glyoxylate shunt is less important. Ser, Gly, Ala, Val, Asp and Glu are *de novo* synthesized efficiently.

Finally, *M. tuberculosis* grown in resting and activated bone-marrow-derived macrophages show substantial upregulation of the type II citrate synthase gene (*gltA*), the isocitrate lyase gene (*aceA1*), the PEP carboxykinase gene (*pckA*) and the malate dehydrogenase gene (*mez*) implying corresponding protein partner complexes. There is good evidence that fatty acids, and possibly glycerol or glycerol-3P, are the preferred carbon sources (β-oxidation is important for virulence), as there is not much amino acid synthesis, and glucose utilization may be confined to early states of infection [1].

#### 2.2.2. Regulatory Strategies and Prokaryotic Protein Complexes

Environmental perturbations, nutrient change or shortage, stress responses and density of individuals all have impact on metabolism. Furthermore, several levels of regulation (transcription, translation, protein stability, enzyme regulation) ensure that the response is optimal. Regarding regulation of RNA and protein complex half-life, experimentally determined mRNA and protein half-lives were measured by Maier *et al.* [30] under different environmental conditions for *M. pneumoniae*. Gene expression was not well correlated with protein dynamics. The translation efficiency was more important for protein abundance than protein turnover. Combining stochastic simulations and *in vivo* data the authors showed that low translation efficiency and long protein half-lives “effectively reduce biological noise in gene expression” [30]. Protein abundances were found to be regulated in functional units and according to cellular state. This included protein complexes and pathways.

Considering regulatory input is far more challenging. A first observation is from Jozefczuk *et al.* [6], studying *E. coli* metabolism and regulatory response after different types of challenges comparing metabolome and transcriptome. The responses to different stimuli vary. However, there is a general strategy of energy conservation. Central carbon metabolism intermediates go down fast if cell growth stops. Summing up the various scenarios, Jozefczuk *et al.* [6] found a condition-dependent association between metabolites and transcripts. Thus, also in *E. coli*, a direct correlation between gene expression and metabolites is only possible for the central carbohydrate pathways glycolysis, pentose phosphate cycle and citric acid cycle [31], otherwise the condition-specific regulation has to be considered (Figure 2). 

Using a combination of computational tools including elementary mode analysis, as well as a new technique involving metabolic flux patterns [32], methods from network inference and dynamic optimization, Wessely *et al.* [33] showed for *E. coli* that transcriptional regulation of pathways reflects the protein investment into these pathways. As an evolutionary optimal strategy, protein-expensive pathways are tightly controlled by many interactions, whereas metabolic cheap ones are not.

Furthermore, niche and species-specific regulatory strategies allow model pathogens for intracellular infections to be optimally adapted to their own niche in the host. Each pathogen uses few specific transcription factors to adapt, which then bind to the promoters together with polymerase and sigma factors leading to transcriptional protein complexes for all the genes they control during the adaptation process [1]: PrfA in *Listeria* is used only for adaptation to nutrient-poor conditions on specific media or in the host. Transcriptional regulators VirF, VirB and MxiE are used in *Shigella*. In contrast pathogenic *Salmonellae* have a more elaborate regulation of their intracellular adaptation exploiting pathogenicity islands and as regulatory components the transcription activator HilA and the SsrAB two-component system. Specific virulence genes are not so clear in *M. tuberculosis*. However, each of the four *mce* operons, which are essential for virulence, is repressed by a specific transcriptional repressor under various nutrient rich cultivation conditions. This repression is presumably relieved when nutrient conditions are sufficiently poor (host cell, macrophage, persistence). 

For comparison, *S. aureus* adaptation to glucose limitation and other environmental challenges involves regulatory switches, again both on the transcriptional level, as well as regarding metabolism (e.g., pyruvate dehydrogenase complex; Figure 2). During the aerobic/anaerobic shift, Rex- (redox sensing) regulators are involved both in redox sensing and in regulation of anaerobic gene expression [34] using a highly conserved binding sequence to repress genes downstream. This improves anaerobic reduction of NAD^+^ to NADH (lactate, format and ethanol formation), nitrate respiration and ATP synthesis. 

A tight connection of metabolism, regulation and coordinated shifts in protein complexes and system states is also observed in other fast growing organisms, such as yeast [35].

#### 2.2.3. A System-Wide, Global View on Prokaryotic Protein Complexes

Given the fact that adaptation of metabolic networks happens in concert involving many pathways and that regulators are rather highly interconnected, an alternative to model bacterial adaptation are more global views. Thus, it is interesting to compare how metabolic changes are coupled to a response. Whereas eukaryotes in general rely more on sensing (external and internal) the environment [36], for bacteria, there is a tighter connection to metabolism [7,33] in order to always achieve optimal the growth rate, including just-in-time ribosome synthesis [37]. Whether this can already be called “adaptive prediction of environmental changes” [38] is a matter of preference. However, these overall strategies clearly differ between prokaryotes and even growth-oriented eukaryotic organisms such as yeast. As a general rule, there is a much higher investment in control and sensing in eukaryotes, whereas metabolic adaptation of bacteria exploits direct regulation and coupling to metabolism. This is supported by data from Kotte *et al.* [7] and Jocefzuk *et al.* [6], as well as a number of specific building blocks included in adaptive structures and complexes such as riboswitches [39,40] and the aforementioned trigger enzymes with their double role to switch from metabolic function (often as members of an enzyme complex) to a direct regulatory function (binary complex, often involving nucleic acids) when substrate levels are low [9]. 

There are a number of coordinated adaptation scenarios for *S. aureus* with detailed, coordinated changes in metabolic enzymes, regulation and dynamics of protein complexes. 

Integration of different data sets facilitates a detailed comparison of how mRNA, protein and metabolite flux correlate. Examples of aerobic glucose limitation of *S. aureus* [41], but also for data from *Listeria* [41], show that central carbohydrate pathways (glycolysis, TCA, pentose phosphate cycle) are strongly turned on during this transition and that gene expression, protein levels and metabolite flux correlate well in general. However, exceptions far away from the correlation line point to selected up- or downregulated enzymes, imply changes in enzyme complexes, too. In contrast, for amino acid metabolism, a linear relation at least between gene expression and metabolite flux provides only a lower bound. In such cases, the enzymes are not operating with maximal activity and thus higher mRNA expression than the theoretically calculated minimal level is observed [41]. A number of broader investigations on correlations tend to support such conclusions [31]. In *E. coli*, enzymes of central metabolism are strongly active and thus the corresponding mRNA level is a good indicator of their activity and correlates well with the strengths of the actual metabolic flux through the enzyme. 

The building blocks of system-switching states are different protein complexes in bacteria, and, on the next, the pathway level; a number of pathways change (exactly those concerned with the adaptation as evolution made sure). This is often achieved by development of highly selective transcriptional activation by transcriptional regulators or polymerase subunits if a broader response is necessary, e.g., prokaryotic stress response and specific sigma factors. However, the system perspective is interesting: If such a system change comes about, system stability and self-stabilizing feedback loops have to be taken over. Instead, the new system state has to enhance itself (by positive feedback loops) and once it took over (a tipping point has been reached, the system is committed to change), stable regulation involves further negative feedback loops (a simple example is that the biological oscillations are controlled accordingly; the basic type is the Van der Pol oscillator; [42]). The switch from aerobic to anaerobic growth in *S. aureus* seems in fact to follow that regime under glucose limitation. One can clearly make out central involved protein complexes (Figure 2) which change, concerted pathway adaptations (e.g., all TCA enzymes and respiration is switched off under anaerobic condition) and initial positive feed-back loops (e.g., when the glycolytic enzymes are activated by glucose and low ATP concentrations) with later supporting negative feedback loops (which stop fast metabolization and lead to the stationary phase, including triggering stress response, suitable sigma factor changes in the transcription complexes and binding to a number of different promotor sequences to coordinate stress responses and connected protein complexes to prevent starvation). 

There are more biochemical details to such adaptations, see e.g., Liang [41] for *S. aureus* glucose limitation experiments under aerobic conditions. Thus, when glucose levels are low in *E.coli*, a phosphorylated form of EIIA (phosphotranferase system enzyme) accumulates. This then activates the enzyme adenylyl cyclase. If levels of cAMP increase, the cAMP will bind catabolite activator protein and both together bind to a promotor sequence on the lac operon. Each of these steps leads to concomitant changes in protein complexes, starting from the phosphotransfer system to carbohydrate metabolizing enzyme complexes. However, as these two examples already show, the sequence of changes depends on the succession of concentration changes, the last example would refer well to a situation where there are high concentrations of glucose and, in the end, there is some lactose available to profit from the switch. The prokaryotic response to changing metabolic conditions is thus condition dependent (see e.g., Jozefcuk [6] for data on *E. coli*). However, our overall current understanding of the involved, fine-tuned regulation and feedback, as well as feedforward, loops is limited. More studies to elucidate the details of such physiological changes in protein complexes and bacterial responses to metabolic changes are clearly needed.

In fact, system switching states occur often fast in bacteria. Whole cascades or even larger networks are rapidly reorganized as the whole network is controlled often by one master regulator. A good example is the pathogenicity switch by the PrfA protein of *Listeria* which simultaneously accomplishes (i) adaptation of a number of virulence pathways, and (ii) reorganization of nutrient utilization, thus facilitating adaptation of *L. monocytogenes* from a more saprophytic to an intracellular lifestyle. Also in *Staphylococci* (and many other bacterial species), such major system changes in metabolism (stress response or growth behavior) are mediated with tight control just by the activation of transcription factors (including repressors such as the Rex family). Other switching states include diauxic shift, glucose limitation under aerobic or anaerobic conditions, differentiation (e.g., biofilm formation) or amino acid limitation. 

In a full “on” state for pathways and networks (e.g., growth on full medium and central carbohydrate metabolism) correlation between gene expression and metabolite flux is high. For not-so-central pathways, gene expression data may provide a lower limit as the metabolite flux can still become higher when enzymes are regulated to be more active. However, for such a system-switching state the correlation in activity for the pathways changed simultaneously is high, as seen both for *S. aureus* [41], as well as in other organisms (e.g., Jozefcuk [6] for *E. coli*). Besides the high correlation between the concerned pathways, there are structural changes in complexes such as pyruvate dehydrogenase complex, central for carbohydrate metabolism to accompany such system changes (see examples above). 

However, the involvement for transcription and regulatory factors, changes in the respective protein complexes, correlated pathway changes and correlation between different data sets also apply to other major system changes such as bacterial differentiation (sporulation, apoptosis) and adaptation in general. All these different system states are clearly emergent behavior, which only can come about in the complete bacterium and as a consequence of the full network of interactions, complexes and super-complexes. Further studies of the exact conditions under which bacterial system states change will not only provide insight into complex systems and emergence in general, but is also at the very root of a better understanding of microbial life. Better insights into the links between metabolism and virulence may also help to treat bacterial infections with new vigor.

### 2.3. Crowding and Substrate Channeling for Metabolic Complexes

**Figure 3:**
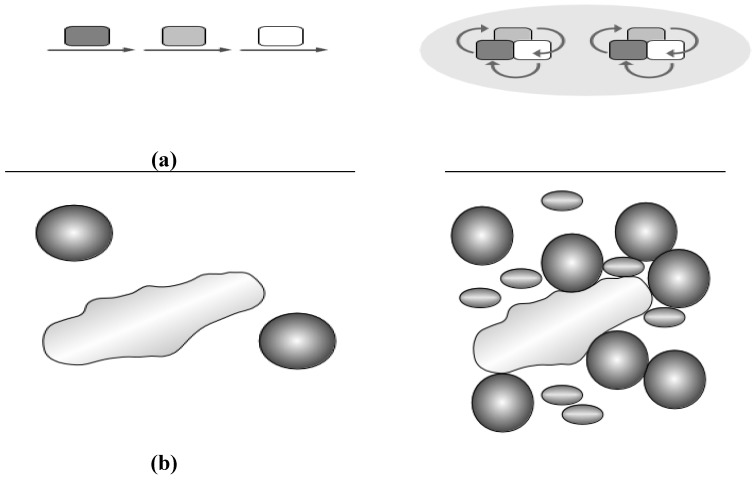
(**a**) Substrate channeling. Originally a conveyor belt concept was invoked (left): Substrate (arrows) is passed from one enzyme to the next (squares in different grey shades). A more modern view (right) considers complexes central for channeling and places the protein complex in the middle, substrates change and are passed around (arrows), furthermore, super-complexes (grey ellipsoid in the background) unite different complexes for even more efficient channeling; (**b**) Molecular crowding. A large molecule (long shape, left) cannot freely move if some obstacles are present (proteins shown as spheres). The effect becomes more pronounced (right) with diverse and more protein species present (different spheres and shades).

On a biophysical level, dynamics of metabolic protein complexes involve also molecular channeling of metabolites, as well as molecular crowding effects (Figure 3).

*Substrate channeling* directly transfers a product to an adjacent cascade enzyme without mixing with the bulk phase, which is again most easily achieved in a static or transient multienzyme complex (Figure 3a). Besides enhanced reaction rates, unstable substrates are protected and metabolic fluxes regulated. Furthermore, this avoids unfavorable equilibria, toxic metabolite inhibition, substrate competition or kinetics [43]. Substrate channeling has also biotechnological potential for metabolic engineering, and cell-free synthetic pathway biotransformation.

Substrate channeling is an old field, started by the Cori’s in the 1950s [44]. Paul Srere coined the concept of “metabolon” to describe improved channeling of substrates in the citric acid cycle [45], encapsulating the concept of what is described here. To study channeling became quite popular in the ‘80s’ (see Tombes and Shapiro [46] on phosphorylcreatinine shuttling; Yang *et al*. [47] on β-oxidation) and ‘90s’ (see Kholodenko *et al.* [48]; Miziorko *et al.* [49] for cholesterol synthesis; Welch and Easterby [50] review a number of different metabolic examples). There is also previous modeling work that shows dynamic channeling is capable of decreasing the metabolite pool sizes (but also able to increase them) [51,52].

Hence, channeling speeds up prokaryote metabolism and involved enzymes. It has already been probed and even changes have been indirectly monitored for carbohydrate metabolism combining a variety of methods: Bauler *et al.* [53] modeled in this way a two-step reaction, using a simple spherical approximation for the enzymes and substrate particles. These authors applied Brownian dynamics to show that spatial proximity and channeling is helpful. Closely aligned active sites are the most effective reaction pathway in their results, but they must not be too close so that the ability of the substrate to react with the first enzyme is not hindered. Protein interaction data comparing *E. coli*, yeast and humans support [54] that indirect protein interactions between related enzymes achieve metabolic channeling. Interestingly, protein complexes include nonenzymatic mediator proteins, sometimes related to signal transduction, to form channeling modules. In *E. coli* reactions, possessing such interactions show higher flux. Channeling could lead to more cross-talk. However, Pérez-Bercoff *et al.* [54] find that scaffolding proteins limit this, keeping protein complexes in separate places. Furthermore, there are interesting differences in the channeling of glucose towards gluconate and other catabolic end-products like pyruvate and acetate, with respect to phosphate status for different Pseudomonas strains (*Pseudomonas aeruginosa versus P. fluorescens*) [55]. Enzyme activities including glucose dehydrogenase, glucose-6-phosphate dehydrogenase and pyruvate carboxylase change in a coordinated fashion in response to changes in growth, glucose utilization or gluconic acid secretion. This includes a shift of glucose towards a direct oxidative pathway under phosphate deficiency which may perhaps also be implied in the different abilities of the two strains to produce gluconic acid. Comparison of enzyme–enzyme interactions in metabolic networks of *E. coli* and *S. cerevisiae* shows evidence for direct metabolic channeling [56]. Enzyme–enzyme interactions occur more often for pathway neighbors with at least one shared metabolite. Non-neighbouring interactions are often regulatory.

*Molecular crowding:* Crowding effects do change prokaryotic enzymes, metabolism and promote protein complexes in prokaryotes. Where metabolic channeling is a specific effect between metabolic proteins (enzymes and protein mediators) in a complex, molecular crowding is instead a more general, unspecific effect by the combined variety of biomolecules (Figure 3b), including nucleic acids, proteins, polysaccharides, as well as other soluble and insoluble components and metabolites (total concentration 400 g/L). The reason for the crowding effect is thus that together these biomolecules occupy a significant proportion (20–40%) of the total cellular volume in cytoplasm and nucleus, respectively [57]. Biophysical effects from crowding differ thus in different compartments of cells. Many nuclear processes such as gene transcription, hnRNA splicing and DNA replication, assemble large protein–nucleic acid complexes. Macromolecular crowding provides a cooperative momentum for these [58], boosting functionally important nuclear activities. In cell membranes, membrane proteins occupy approximately 30% of the total surface area leading to crowding effects on the surface as well as unique effects for the even more movement restricted integral membrane [58]. Thus Wang *et al.* [59] directly monitored the effect of strong crowding on pressure-induced reduction of unfolding of a protein (staphylococcal nuclease) by tryptophan fluorescence. 

Besides such unspecific crowding effects, there are also complex-promoting activities from proteins from the milieu. Thus, in NMR studies, it was observed that high molecular weight glycoproteins are efficient molecular seeds for protein aggregation [60]. Such additional effects were also invoked by McGuffee and Elcock [61], using a simulation model which successfully describes the relative thermodynamic stabilities of proteins measured in *E. coli*, modeling 50 highly abundant macromolecule types at experimentally measured concentrations. Morelli *et al.* [62] show a simple way to model the effects of macromolecular crowding on biochemical networks. To succeed, they had to scale bimolecular association and dissociation rates correctly. They used kinetic Monte Carlo simulations and looked at crowding effects, comparing a constitutively expressed gene, a repressed gene, and a model for the bacteriophage λ genetic switch. Each molecular assembly was modeled both with and without nonspecific binding of transcription factors to genomic DNA. Furthermore, crowding effects shifted association–dissociation equilibria rather than slowing down protein diffusion, which sometimes had unexpected effects on biochemical network performance. Norris and Malys [63] show even changes of Michaelis-Menten kinetic constant *K_m_*, and rate constant *k_cat_* for the enzyme glucose-6-phosphate dehydrogenase under crowding. *k_cat_* increased at very low concentrations of crowding agent or at high crowded concentrations during heating (45 °C), adding PEG. Simulations applying the Arrhenius equation agree with these observations. 

More subtle effects of how enzymes are influenced by crowding are apparent in simulations and only partly supported by experimental data: Adenylate kinase was coarse grain modeled by Echeverria and Kapral [64], showing large-scale hinge motions during enzymatic cycles. Multiparticle collision dynamics included effects due to hydrodynamic interactions. A stationary random array of hard spherical objects provided crowding in the simulation. Adenylate kinase prefers a closed conformation for high volume fractions (smaller obstacle radius and tighter packing). Average enzymatic cycle time and characteristic times of internal conformational motions of the protein change, as do the transport properties. Under crowding, diffusive motion becomes up to ten times slower with longer orientational relaxation time. In general, and according to simulations on seven different proteins, those experiencing the strongest crowding effects have larger conformational changes between open and closed states [65]. In Brownian dynamic simulations, Ando and Skolnick [66] modeled a simplified *E. coli* cytoplasm with 15 different macromolecule types at physiological concentrations and sphere representations using a soft repulsive potential. These authors compare their data with the experiment; at cellular concentrations, the calculated diffusion constant of GFP was shape independent and much larger than in the experiments. However, including hydrodynamic interactions using the equivalent sphere system reproduced the *in vivo* experimental GFP diffusion constant without further parameter adjustment. Nonspecific attractive interactions reduced strongly diffusivity of the largest macromolecules [66]. The authors observed attractive clusters around these, but not if hydrodynamic interactions dominated. The latter led also to size-independent intermolecular dynamic correlations. Both models are interesting, and the noted differences between both models should now be directly compared to further experimental data. Even the change in the binding free energy due to crowding could be quantitatively described by the scaled particle theory model without any fitting parameters [67]. Crowders of different sizes were predicted by the same model with an additive setup. Crowding increased the fraction of specific complexes and nonspecific transient encounter complexes were reduced in a crowded environment as the nonspecific complexes had greater excluded volume [67]. However, more experimental data are needed to confirm these detailed predictions.

## 3. Conclusions

Metabolic adaptation in prokaryotes is efficient and involves a number of different protein complexes, many of them changing rapidly as metabolic conditions change. Our description of protein complexes and metabolism combines large-scale studies with bioinformatics approaches and individual experiments. Conditions in the prokaryotic cell correspond to a tightly packed hyper-complex and it has become clear that a biophysics dominated by metabolite channeling and crowding is important to understand prokaryotic metabolism and efficiency of involved protein complexes and enzyme ensembles. Overall knowledge on protein complexes is good for several model organisms. However, regarding specific complexes and their changes, many details are still to be discovered. This includes more insights on trigger enzymes, super-complexes, as well as links between regulation, adaptor proteins and enzyme chains. A systems biology perspective helps to integrate these different aspects on protein complexes into the context of metabolic adaptation in prokaryotes.
